# Cost-Effectiveness of Vaccinating Immunocompetent ≥65 Year Olds with the 13-Valent Pneumococcal Conjugate Vaccine in England

**DOI:** 10.1371/journal.pone.0149540

**Published:** 2016-02-25

**Authors:** Albert Jan van Hoek, Elizabeth Miller

**Affiliations:** 1 Department of Infectious Disease Epidemiology, London School of Hygiene and Tropical Medicine, Keppel street, WC1E 7HT, London, England; 2 Immunisation, Hepatitis and Blood Safety Department, Public Health England, 61 Colindale Avenue, NW9 5EQ, London, England; Centers for Disease Control & Prevention, UNITED STATES

## Abstract

**Background:**

Recently a large clinical trial showed that the use of 13-valent pneumococcal conjugate vaccine (PCV13) among immunocompetent individuals aged 65 years and over was safe and efficacious. The aim of this study was to assess the cost-effectiveness of vaccinating immunocompetent 65 year olds with PCV13 vaccine in England. England is a country with universal childhood pneumococcal conjugate vaccination programme in place (7-valent (PCV7) since 2006 and PCV13 since 2010), as well as a 23-valent pneumococcal polysaccharide (PPV23) vaccination programme targeting clinical risk-groups and those ≥65 years.

**Method:**

A static cohort cost-effectiveness model was developed to follow a cohort of 65 year olds until death, which will be vaccinated in the autumn of 2016 with PCV13. Sensitivity analysis was performed to test the robustness of the results.

**Results:**

The childhood vaccination programme with PCV7 has induced herd protection among older unvaccinated age groups, with a resultant low residual disease burden caused by PCV7 vaccine types. We show similar herd protection effects for the 6 additional serotypes included in PCV13, and project a new low post-introduction equilibrium of vaccine-type disease in 2018/19. Applying these incidence projections for both invasive disease and community-acquired pneumonia (CAP), and using recent measures of vaccine efficacy against these endpoints for ≥65 year olds, we estimate that vaccination of a cohort of immunocompetent 65 year olds with PCV13 would directly prevent 26 cases of IPD, 69 cases of CAP and 15 deaths. The associated cost-effectiveness ratio is £257,771 per QALY gained (using list price of £49.10 per dose and £7.51 administration costs) and is therefore considered not cost-effective. To obtain a cost-effective programme the price per dose would need to be negative. The results were sensitive to disease incidence, waning vaccine protection and case fatality rate; despite this, the overall conclusion was robust.

**Conclusions:**

Vaccinating immunocompetent individuals aged ≥65 years with PCV13 is efficacious. However the absolute incidence of vaccine-type disease will likely become very low due to wider benefits of the childhood PCV13 vaccination programme, such that a specific PCV13 vaccination programme targeting the immunocompetent elderly would not be cost-effective.

## Background

Community acquired pneumonia (CAP) causes a high disease burden among the aging population. The bacterium *Streptococcus pneumoniae* is the single most common bacterial cause of CAP, with an estimated 5%-40% of cases caused by this pathogen [1–3]. Furthermore, *S*. *pneumoniae* causes invasive disease (IPD) associated with a high mortality depending on age and risk-group [4]. For this reason many developed countries have introduced a pneumococcal vaccination programme targeting those aged 65 years and over using the 23-valent polysaccharide vaccine (PPV23) which covers 23 of the 90+ known pneumococcal serotypes. Unfortunately, due to a relatively low efficacy and short duration of protection against IPD, and lack of protection against pneumococcal-attributable CAP, PPV23 has had little overall impact on pneumococcal disease in the 65+ age group. [5–7].

Recently the results of a large clinical trial [clinical trial registration number NCT00744263] in the Netherlands showed that the 13-valent pneumococcal conjugate vaccine (PCV13) currently used in the childhood vaccination programme of many countries has an efficacy of 45.6% (95% CI 21.8%-62.5%) against vaccine-type CAP and 75% (95% CI 41.4%-90.8%) against IPD among those aged 65 years and older [8].

In this study we investigate the cost-effectiveness of adding PCV13 to the current PPV23 vaccination schedule targeting those aged 65 years and over in England. To make a realistic projection of the future incidence of vaccine-type CAP and IPD in this age group, the herd protection effects from the childhood pneumococcal vaccination programme, in which the 7-valent PCV (PCV7) has been recommended for all infants since September 2006 and PCV13 since 2010, were taken into account.

## Methods

### Programme

In this study we investigate the cost-effectiveness of offering PCV13 to all 65 year olds in England. This would be an addition to the current PPV23 programme in which a dose of PPV23 is offered to any 65 year old who has not previously received a dose at any time in the past. To make a realistic projection of the future incidence of vaccine-type CAP and IPD in this age group, the herd protection effects from the childhood pneumococcal vaccination programme, in which the 7-valent PCV (PCV7) has been recommended for all infants since September 2006 and PCV13 since 2010, were taken into account. It is assumed that PCV13 will be provided at the same time as seasonal influenza vaccine, but that PPV23 will need an additional visit to the general practitioner (GP) eight weeks later. Therefore the new programme will require one extra GP visit compared to the current schedule. The first season of use was set to be 2016/2017.

### Study Population

The study population was set to be a cohort of people who reflect the targeted population of the clinical trial. This cohort comprised immunocompetent individuals including those with co-morbidities such as diabetes, asthma, splenectomy or heart, liver or lung disease. The clinical trial excluded those living in nursing homes or long term care facilities but due to lack of data on pneumococcal disease burden in these groups in England it was not possible to exclude such individuals from analysis.

### Incidence of IPD

The incidence of IPD by vaccine-type (PCV7 and PCV13 minus 7) was based on the serotype- specific surveillance data collated by Public Health England for the epidemiological years July to June from 2002/03 to 2013/2014. Details of these incidence estimates for England and Wales have been recently published by Waight et.al. [9]. We corrected for changes in surveillance sensitivity over time by increasing the incidence of IPD before 2009/10 as previously described [9].

### Incidence of CAP

Recently Rodrigo and colleagues [10] published a survey of community acquired pneumonia in two large teaching hospitals in Nottingham (UK). Over a period of five years (September 2008 until September 2013) urine samples were collected from participants (aged ≥16 years) following admission with acute lower respiratory symptoms. Samples were tested with a validated multiplex immunoassay (detecting 14 serotypes/groups, including all PCV 13 types) and the immunochromatographic assay kit BinaxNOW^™^ (Alere) which detects pneumococcal polysaccharide. In addition, blood samples were cultured for *S*. *pneumoniae* and positives serotyped by slide agglutination. Annual incidences for vaccine types (PCV7 and PCV13 minus 7) were estimated using the population data from the Office of National Statistics for the greater Nottingham area. To our knowledge this is the largest longitudinal survey that documents the impact of PCV vaccination on vaccine-type pneumococcal pneumonia in the UK, hence we used the observations from this study in our projections (see also S1 Appendix).

### Projecting Future Incidence

To estimate the future incidence for IPD and CAP caused by the PCV7 and PCV13 minus 7 vaccine types it was assumed that:

- PCV7 types reached a new post-vaccination equilibrium in IPD in 2013/14.
- The additional 6 types covered by PCV13 will experience a similar reduction in IPD as the PCV7-types, with a similar post-vaccination steady state. (see S2 Appendix for evidence in support of this assumption)
- The incidence of PCV13-VT pneumococcal CAP will follow a similar downward trend as IPD.

To implement part b the IPD incidence rate ratio for PCV7 types was calculated for the period since introduction of PCV-7 (2006/07 until the most recent data) using the pre-PCV7 period 2002/03-2005/06 as baseline incidence. Subsequently this incidence rate ratio was used to project the IPD incidence for 2014/2015 onwards for the PCV13 minus PCV7 types, using the pre-PCV13 incidence in the years 2007–2011 as baseline. To project the incidence of PCV7 and PCV13 minus 7 CAP as described in part c, the ratio between CAP and IPD was calculated by ordinary least squares for the period 2008–2013, the years for which overlapping data was available. Subsequently this ratio was used to predict the future incidence of CAP based on the projected IPD incidence.

### Vaccine Efficacy and Coverage

In the clinical trial population (per protocol analysis) the protection was 75% against PCV13-type IPD and 45.6% against PCV13-type CAP [8]. For CAP there were also age-stratified results: 52.4% (95% CI 24.1%-71.0%) protection among those aged under 75 and 46.4% (95% CI -4.3%-73.6%) for those age ≥75 and <85, and no efficacy among those age 85 years and over (6 cases in vaccine arm vs 3 in the placebo arm) [8]. However, in a programmatic setting the intention-to-treat analysis is more applicable, as the vaccinated cohort will include those who develop immunocompromised conditions. Unfortunately no age specific estimates were available using the intention-to-tread analysis, but as the overall estimate was lower (37.7%) the non-age specific estimate of 45.6% was applied, being a compromise between the younger age and the inclusion of those who will develop immunocompromised conditions. The average duration of follow-up in the trial was almost 4 years, and in these four years there was not an obvious decline in protection (although there was simultaneously a decline in the force of infection of some PCV13 vaccine types due to the herd immunity impact of the 10–valent PCV childhood vaccination programme introduced in the Netherlands in 2011 and which covered the same serotypes as PCV13 apart from 3, 6A and 19A.) [8]. Given the limited duration of follow up in the trial population we did not assume lifelong protection from PCV13. To be conservative we used a waning scenario developed by the manufacturer Pfizer which assumes a constant protection for the first 9 years after which it drops every 5 years until there is a constant protection from 20 years onward (Cost-effectiveness analysis of adult vaccination with the 13-valent Pneumococcal Conjugate Vaccine in the United Kingdom, unpublished report provided to the authors by Pfizer). The protection against IPD was respectively 75% (year 1–9), 43% (year 10–14), 9% (year 15–19), and 5% (years 20+); for CAP this was 45%, 26%, 5% and 3%. The vaccine uptake was set to be 69%, in line with the uptake of the PPV23 vaccine in those aged 65 years and over [11].

### Mortality, Life Expectancy and Quality of Life

The loss in quality of life due to pneumococcal disease was similar to that applied in Rozenbaum et al [12], a previous cost-effectiveness study on the use of PCV13 in high risk groups including those aged ≥65 years in the UK conducted before the clinical trial results became available and which assumed in its base case no protection from PCV13 against vaccine-type CAP. For IPD various sequelae from meningitis were included and adjusted for the fact that one person can have multiple sequelae at the same time. The assumed rate for meningitis was 6% [12] and the assumed duration of sequelae was lifelong. The overall QALY loss due to IPD was between 0.14 (aged 65) and 0.01 (aged 100) depending on age. The assumed QALY loss due to CAP was set on 0.006 [12]. The case fatality rate (CFR) was assumed to be 30% and 10% for IPD and CAP respectively. Both parameters are uncertain; the IPD CFR was based on a study in which laboratory confirmed IPD data was linked to hospital records to ascertain deaths during an IPD admission [4]; the CFR for CAP was set as a compromise, as various sources report great differences in rates, from 1.8% in the CAPITA trial [8], 6.2% in the Rodrigo study and over 20% based on computerised hospital discharge data for admissions with a code for all cause pneumonia [13]. The effect of varying the CFR for IPD and CAP was investigated in a sensitivity analysis. The quality adjusted life expectancy was estimated using the most recent mortality rates [14] and the background quality of life was as estimated by Petrou [15]. A scenario analysis was performed using a longer life expectancy estimated for a cohort of non-risk people (excluding all clinical risk groups recommended to receive PPV23 under the age of 65). Persons could not become older than 105, hence the maximum follow-up for a 65 year old was 40 years.

### Costs

The cost of hospitalisation due to IPD and CAP was based on Rozenbaum et al. [12] and inflated to 2014 costs using the Hospital & Community Health Service Index [16]. The price for IPD was between £4865 (age 65) and £4780 (age 100) and £715 for CAP. The current price of PCV13 in the British National Formulary is £49.10 [17] and this price was used in the base case. The administration costs due to the additional dose was set to be £7.51.

### Economic Model

A static cohort model was used to estimate the future incidence, cost and disease burden for specific ages. In the model cohorts were followed from their point of entry in the season of 2016/2017 until death. The incremental cost-effectiveness ratio was calculated from a health care payer’s perspective and cost and QALYs were both discounted by 3.5% per annum.

To achieve an understanding of the relation between the cost-effectiveness ratio and vaccine price we performed various sensitivity analyses to test the cost-effectiveness ratio with a vaccine price of £49.10 and the price at which the vaccine becomes cost-effective using a threshold of £20,000 as is recommended in the UK [18]. The robustness of the outcome was tested by varying the assumptions on costs, QALY loss, case fatality rate, incidence, waning of vaccine protection, age at first dose, life expectancy, timing of introduction.

The decision taken by the UK Joint Committee on Vaccination Immunisation on the use of PCV13 in those aged 65 years and over was split into two steps; first a decision on the use of PCV13 in the general (immunocompetent) population and secondly a decision on the use of the vaccine in clinical risk-groups. In the presented base-case analysis the immunocompetent population includes people in clinical risk-groups; therefore we have added a scenario which focuses on the cost-effectiveness among those who have no underlying co-morbidity. The incidence presented in Waight et al. [9] is for all individuals irrespective of co-morbidities. To adjust this population incidence to reflect those who are not in a clinical-risk group we assumed that 55% of the population aged 65 years and over is not in a high risk group [4] and that the odds ratio of hospitalisation if in a risk versus a non-risk group is 2.7 [4]. This implies that the incidence would be 55% lower in the “non-risk” group. (See S3 Appendix)

## Results

**Fig 1:**
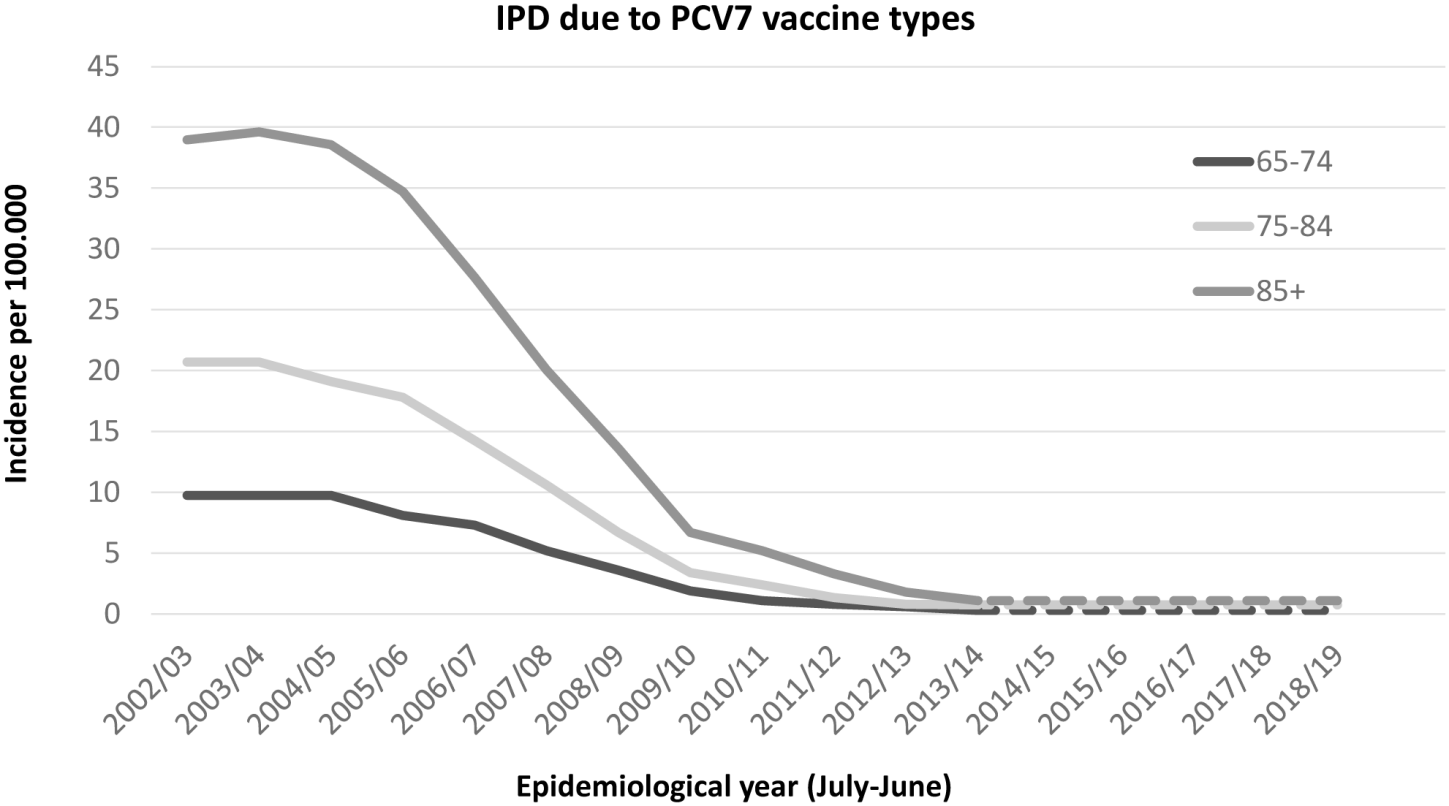
Observed and projected incidence for invasive pneumococcal disease caused by the PCV7 vaccine types per 100.000 persons for the epidemiological years 2002/03 until 2019/20 in three age groups 65–74 (black), 75–84 (light grey) and 85 and over (dark grey line). Future projections, see text, are shown by a dotted line.

**Fig 2:**
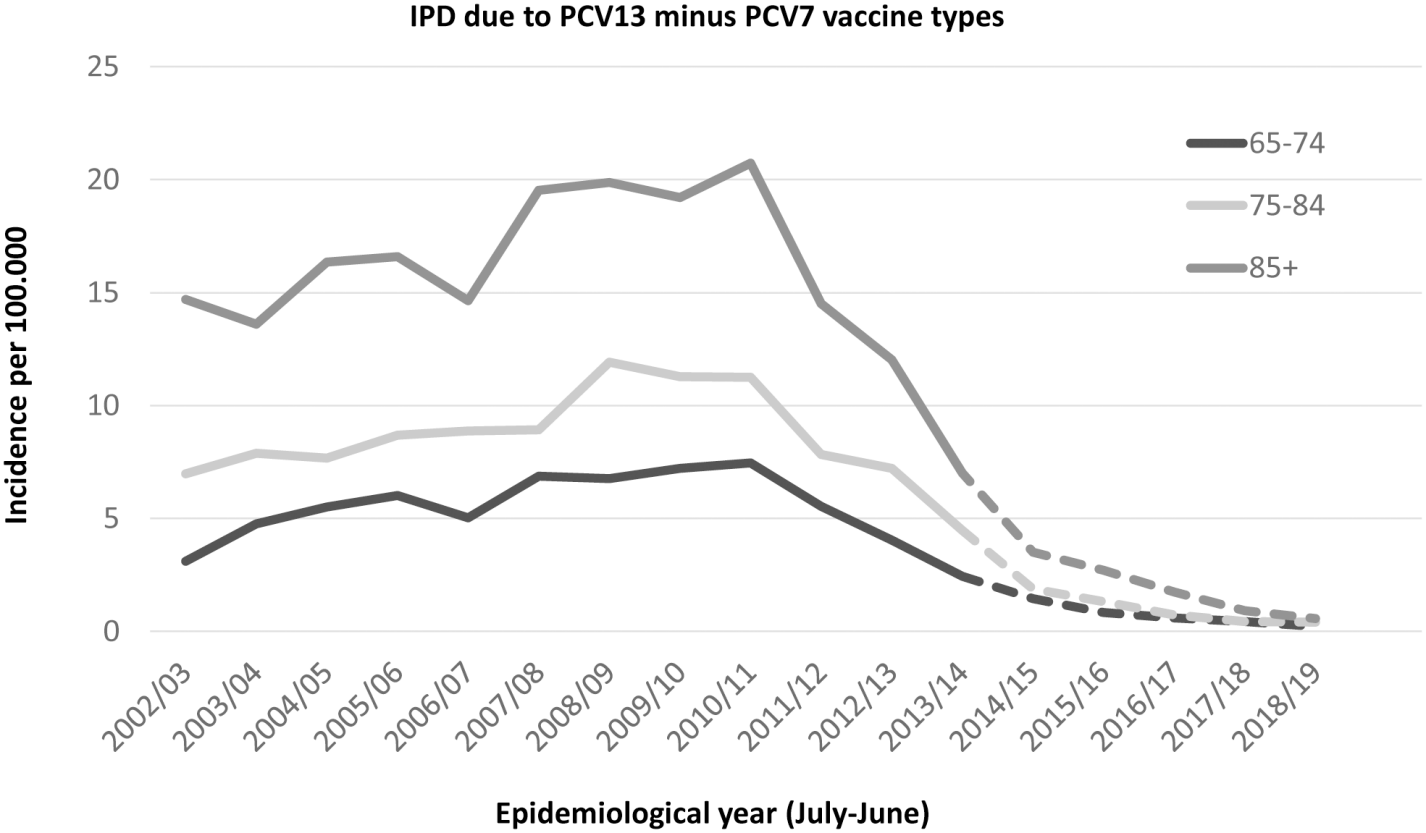
Observed and projected incidence for invasive pneumococcal disease caused by the PCV13 minus PCV7 vaccine types per 100.000 persons for the epidemiological years 2002/03 until 2019/20 in three age groups 65–74 (black line), 75–84 (light grey line) and 85 and over (dark grey line). Future projections, see text, are shown by a dotted line. The incidence in 2018/19 and 2019/20 will continue into the future.

**Fig 3:**
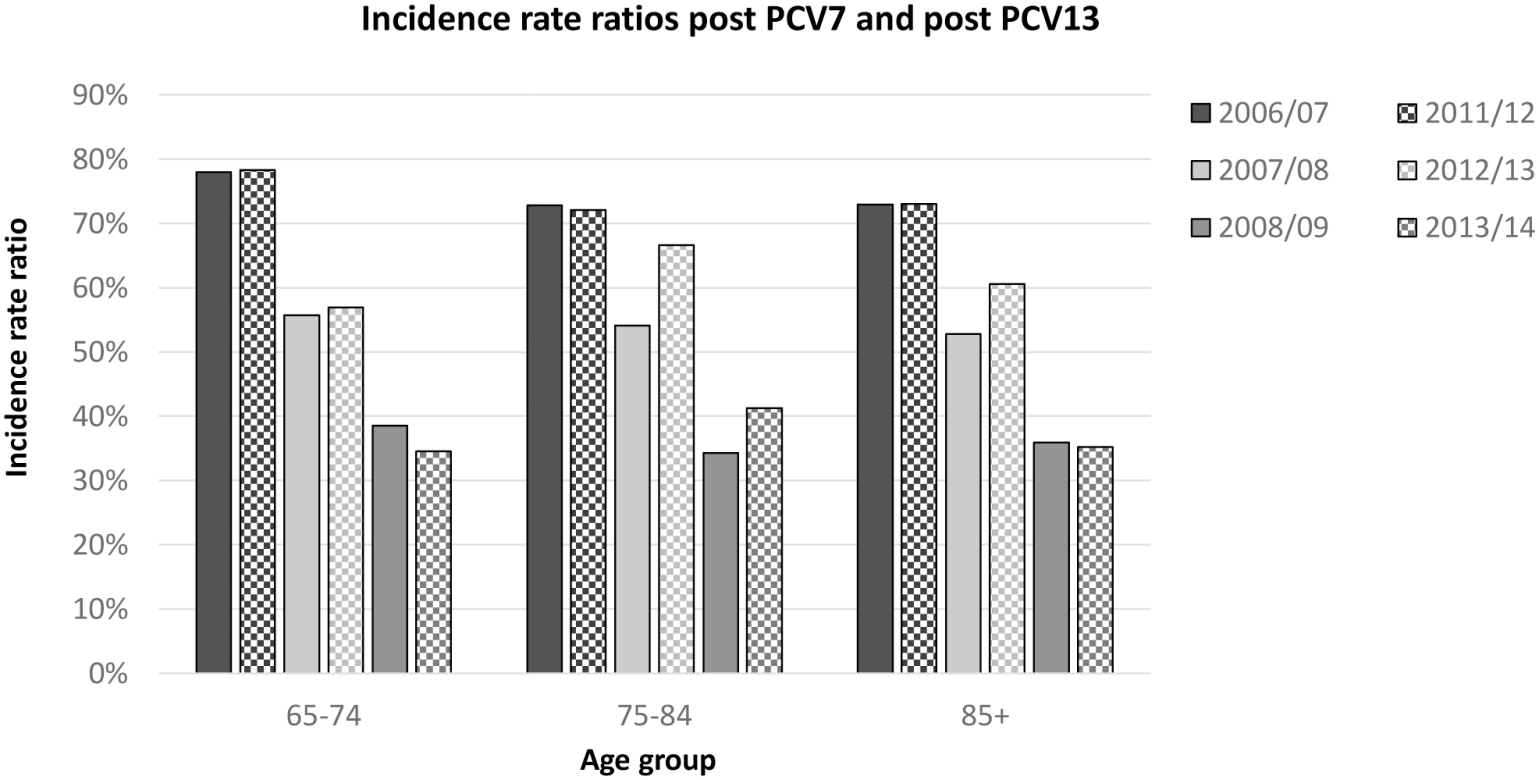
Observed incidence rate ratios for PCV7 vaccine types and PCV13 minus PCV7 serotypes in the three years after vaccination compared to the four years before vaccination (2002/03 to 2005/06 for PCV7 and 2007/08 to 2010/2011 for PCV13 minus PCV7). The dark shaded bars represent PCV7 and the light shaded bars PCV13 minus PCV7 types.

**Fig 4:**
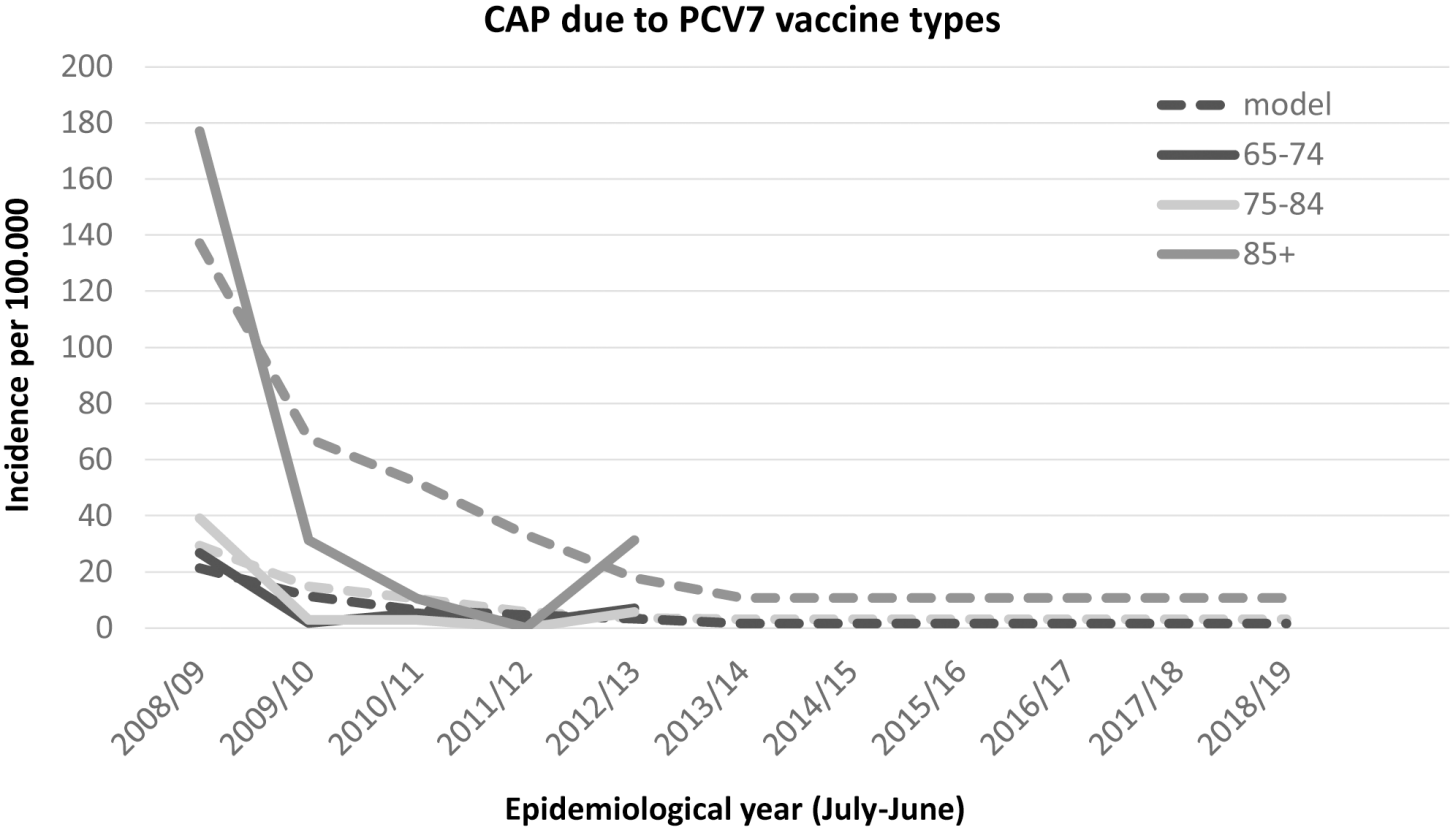
Observed and projected incidence for community acquired pneumonia caused by PCV7 vaccine types per 100.000 for the epidemiological years 2008/09 until 2019/20 in three age groups 65–74 (black line), 75–84 (light grey line) and 85 and over (dark grey line). Model projections, see text, are shown by a dotted line. The incidence in 2018/19 and 2019/20 will continue into the future.

**Fig 5:**
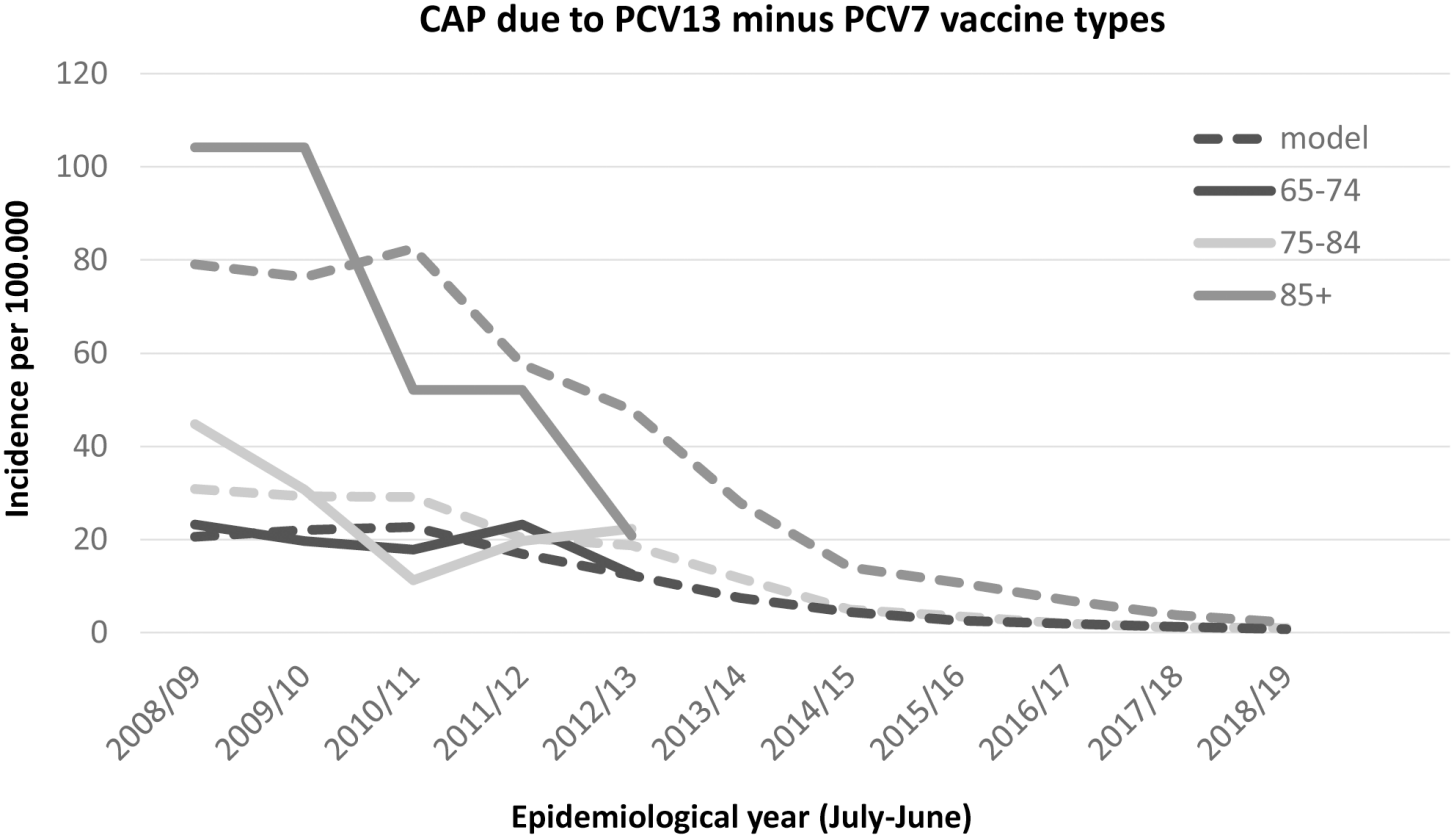
Observed and projected incidence for community acquired pneumonia caused by PCV13 minus PCV7 vaccine types per 100.000 for the epidemiological years 2008/09 until 2019/20 in three age groups 65–74 (dark line), 75–84 (light grey line) and 85 and over (dark grey line). Model projections, see text, are shown by a dotted line. The incidence in 2018/19 and 2019/20 will continue into the future.

The observed (and projected) incidence of invasive disease due to PCV7 serotypes has become very low, with an incidence below or around 1 per 100,000 (depending on age), with the highest incidence among those age 85 years and over (see Fig 1). The incidence rate ratio compared to the pre-vaccine period is around 3% (Table 1). The projected incidence for PCV13 minus PCV7 types is expected to reach its steady state in the season 2018/19 after when it is assumed that the incidence will remain low (Table 2, Fig 2). Due to the higher circulation of PCV7 types before introduction of the vaccine it is projected that in the long term more disease will be caused by PCV7 types than the additional 6 serotypes in PCV13. The incidence rate ratio for both the PCV7 and PCV13 minus PCV7 is similar using the first three post-vaccination years (see Fig 3). The incidence of CAP due to PCV7 and PCV13 minus 7 serotypes was projected to reduce to low incidences in line with the reductions in IPD (Table 2, Figs 4 and 5 respectively). Overall, an expanding herd immunity effect induced by the childhood vaccination programme is observed, resulting in low incidences for vaccine type disease (both IPD and CAP) in those age 65 and over.

**Table 1:** Estimated IPD to CAP incidence rate ratio over the period 2008/09 to 2012/2013.

Age group	PCV7	PCV13 minus PCV7
65–74	5.9	3.0
75–84	4.4	2.6
85+	10.1	4.0

**Table 2:** Projected future incidence of Invasive pneumococcal disease (IPD) and Community Acquired Pneumonia (CAP) caused by the PCV13 vaccine types per 100,000. In 2018/2019 a new steady state was assumed to be reached and the same incidence was continued after.

	2015/2016	2016/2017	2017/2018	2018/2019
Projected IPD caused by PCV13-types				
65–74	1.10	0.88	0.71	0.49
75–84	2.04	1.44	1.15	1.10
85+	3.76	2.80	1.99	1.62
Projected CAP caused by PCV13-types				
65–74	4.17	3.50	2.95	2.31
75–84	6.55	4.99	4.24	4.13
85+	21.45	17.59	14.40	12.93

Using the projected incidences by age, by year, and the assumptions in the cost-effectiveness model (Table 3) it is possible to project the disease burden in a cohort of 442,435 65 year olds until death from 2016 until 2056 with a vaccine uptake of 69%. Using the projection it is estimated that there will be 82 cases of vaccine type IPD and 426 cases of vaccine type CAP and a total of 67 deaths in a cohort of 442,435 65 year olds, linked to a total cost of just over £0.7 million (£0.4 million discounted) and 367 QALYs lost (257 discounted). With a vaccination programme this will be 56 cases of IPD and a remaining 357 cases of CAP and a total of 52 deaths, and £0.5 million costs (£0.3 million discounted) and 257 QALYs (160 discounted).

**Table 3:** Assumptions in the base case cost effectiveness analysis.

Parameter	IPD	CAP
*Vaccine efficacy*		
Year 1–9	75%	45%
Year 10–14	43%	26%
Year 15–19	9%	5%
Years 20+	5%	3%
*Costs*		
Hospitalisation (incl. sequelae)	£4858-£4780 (age dependent)	£715
*Case fatality rate*		
CAP	30%	10%
*QALY loss*		
CAP	0.13–0.01 (age dependent)	0.006
Discounting	Costs	QALYs
Costs	3.5%	3.5%

Assuming a vaccine price of £49.10 per dose the estimated cost per gained QALY is £257,771 and the maximum price to achieve an ICER of £20,000 lies below zero (-£2.83) per dose (on top of the administration costs) (Table 4) The outcome is not very sensitive to the assumed costs and QALY loss for acute disease; however, it is very sensitive to the CFR, waning vaccine-induced protection, as well as the projected incidence of IPD. The optimal age of vaccination is 75 years due to the waning protection and the increase in incidence with age. Even assuming that incidence of PCV13-type IPD and CAP reduce no further after 2015/16, the cost per QALY averted is £145,146, and the maximum price to achieve an ICER of £20,000 is £0.79 (see Table 5).

**Table 4:** Results of the cost effectiveness analysis according to incremental cost effectiveness ratio (ICER) and maximum price per dose; base case and sensitivity analysis.

	ICER using £49.10 per dose and £7.51 administration costs)	Max price per dose (after subtracting £7.51 administration costs) using a threshold of £20,000 per QALY
Base case	£257,771	-£2.83
*Sensitivity analysis*		
Double hospital costs	£256,431	-£2.53
0.05 QALY loss both IPD CAP	£249,357	-£2.68
15%/5% CFR IPD/CAP	£512,829	-£5.00
30%/15% CFR IPD/CAP	£209,423	-£1.82
Age 70	£268,787	-£2.94
Age 75	£262,316	-£2.74
Age 80	£322,910	-£3.53
No waning	£169,638	-£0.29
No back ground QALY loss	£191,863	-£1.33
Extra long life expectancy	£228,661	-£2.27
Double the CAP incidence measured by Rodrigo etal.	£175,664	-£0.71
Long term equilibrium (≥2018)	£287,060	-£3.30
Incidence 55%, to reflect no risk	£469,861	-£4.93

**Table 5:** Incidence of vaccine-type IPD and CAP as applied by year in the model, and the cost-effectiveness and maximum price per dose (threshold of £20,000) for the corresponding incidence in case this would be the long-term incidence.

	2013/2014	2014/2015	2015/2016	2016/2017	2017/2018	2018/19
*Vaccine type IPD*						
65–74	2.7	1.7	1.1	0.9	0.7	0.5
75–84	5.2	2.6	2.0	1.4	1.1	1.1
85+	8.1	4.6	3.8	2.8	2.0	1.6
*Vaccine type CAP*						
65–74	9.1	6.1	4.2	3.5	3.0	2.3
75–84	14.7	8.0	6.5	5.0	4.2	4.1
85+	38.5	24.7	21.5	17.6	14.4	12.9
*Base case scenario*						
ICER	£60,664	£98,767	£145,146	£181,667	£222,378	£287,060
Price/dose	£12.10	£4.63	£0.79	-£0.87	-£2.08	-£3.30
*Scenario restricted to non-risk group population*						
ICER	£98,896	£160,491	£234,824	£293,834	£359,560	£462,838
Price/dose	£4.55	-£0.05	-£2.39	-£3.42	-£4.17	-£4.91

## Discussion

In this analysis we investigated a scenario in which there is a residual burden of vaccine type IPD and CAP despite the herd immunity effects of the paediatric PCV programme. In the base case, and under the most conservative assumption that there will be no further reduction in PCV13 type IPD and CAP after 2015/16, vaccination of the immunocompetent elderly is very unlikely to be cost-effective under the current list-price (£49.10). Due to the additional GP visit and the related administration costs when both PPV23 and PCV13 are used the budget which remains available to purchase the vaccine translates into a cost of below £0 per dose in the base case scenario. Even if the administration cost was zero, the price per dose would need to be below £5.

There are several uncertainties in our assessment. The first is the applied future incidence for both IPD and pneumococcal CAP. In our analysis we investigated a base case where the future was based on the observed reduced rate of IPD caused by PCV7 serotypes in those aged 65 years and over as the result of the PCV7 and PCV13 vaccination programmes for infants. This rate reduction was applied to the remaining serotypes covered by PCV13 in invasive disease for future years, and then the incidence of pneumococcal CAP was extrapolated from the trend in IPD. We assumed for the base case that the 97% reduction in PCV7 type IPD observed in both 2012/13 and 2013/14 in those aged 65 years and over represents the level at which PCV7 type IPD will remain in the future and that the reduction in PCV13 minus 7 type IPD will follow a similar trajectory after the change to PCV13 but with a delay of a year relative to the PCV7 herd effect due to the lack of a catch-up programme. The observed similarities in rate reduction between PCV7 and PCV13 for the three years for which there is similar data supported the use of the decline among PCV7 types to project the decline among PCV13 types. The extrapolation of disease trends in IPD towards pneumococcal CAP is justified for two reasons; firstly a similar proportionate reduction in both IPD [9] and pneumococcal CAP [10] was shown in the post-vaccination period in England (S2 Appendix) suggesting a herd protection effect by the childhood vaccination programme. Secondly, this herd protection effect can only originate from a reduced transmission of the vaccine-types in the population. This accords with the lower carriage rates for vaccine-type serotypes among non-vaccinated cohorts shown in England for both PCV7 [19] and PCV13-7 types [20]. As both IPD and pneumococcal CAP incidence depend on the transmission of vaccine-types, similar trends are expected in both disease presentations. A second uncertainty is the overall health benefit of the vaccination programme on prevented mortality. In this there are three factors: these are 1) the risk of death given CAP or IPD, 2) the future life expectancy of a person if they would not have died and 3) the quality of life this person would have enjoyed for the remaining life time. The CFR rate for IPD was based on a 30-day mortality in hospital among laboratory confirmed cases, without an assessment of causality, therefore this is likely to be an overestimation. The CFR due to CAP is not clear. Among the 185 identified cases of pneumococcal CAP in the CAPiTA trial there were only 7 deaths, corresponding to a mortality rate of 3.8% (and only 2 out of 112 VT CAP = 1.8%). In the pneumonia study from Nottingham used for the incidence of vaccine type CAP in this study the mortality was 8.2% in the total cohort (all cause CAP) and 6.3% among those with pneumococcal CAP. A recent meta-analysis of mortality in 23 studies of the outcome of CAP showed that the overall CFR was 4.3% in randomised controlled trials and 5.5% in observational studies; the total number of subjects included in the meta-analysis was 137,574, and the mean ages of participants ranged from 59 to 79 years across the studies [19]. This shows that the 10% CFR for CAP used in the base case is likely to be a high estimate. The life expectancy used is the life expectancy observed in the overall population, but in the sensitivity analysis it was shown that using a longer life expectancy did not influence the outcome much. In cross-sectional surveys of the quality of life in the general population older people score lower, as was included in our analysis. However, even if this background decrement in quality of life with age is ignored, a PCV13 programme for 65 year olds would not be cost effective. A third uncertainty is the duration of protection. This is a major uncertainty in the overall cost-effectiveness for this vaccine, and without additional data will remain speculative. However, the base case scenario with no decrement for the first 10 years is likely to be conservative. It is important to match the study population with the population in the clinical trial from which the vaccine estimates are derived. Although the CAPiTA trial excluded severely immunocompromised people it did include most people with co-morbidities (only 0.3% of recruited people were excluded based on clinical criteria). Therefore we decided to use the IPD and CAP incidence in the general population but performed a sensitivity analysis in which the incidence was lowered and the life expectancy was increased. This only reduced the cost-effectiveness and decreased the cost effective price for the vaccine. The costs and QALY loss were based on a previous study but remain uncertain parameters. In the sensitivity analysis however we show that these assumptions did not influence the overall outcome much.

A recent review of cost-effectiveness studies addressing the same question [21] found that 9 out of the 10 available analysis considered vaccination of those 65 and older with PCV13 cost-effective. However, most of these studies were performed before the publication of the clinical trial results, and other assumptions, such as the assumed herd immunity effect of a childhood immunisation programme on the vaccine-type disease burden in other age groups, were not always informed by robust evidence. The one study included in the review with similar findings was our previous study in which showed that vaccinating clinical risk-groups in England with PCV was not cost-effective [12]. Furthermore, a more recent cost-effectiveness study in the Netherlands [22] found that it is not cost-effective to vaccinate immunocompetent elderly aged between 65 and 74 years with PVC13. Interestingly the latter was under a scenario with a 10-valent childhood vaccination programme in place after several years of PCV7, in the study it was assumed that the PCV10 programme would have no indirect effects on the 3 additional serotypes included in PCV10 and no cross-protection against the 3 additional serotypes included in PCV13. They investigated however a medium-risk group of persons who are immunocompetent but have underlying conditions, as it is programmatically difficult to identify these people this risk-group was not included in this analysis.

The finding of this study supported the decision of the Joint Committee for Vaccination and Immunisation (JCVI) [to be cited] that PCV13 will not be universally recommended for those aged 65 years and over in England. This in contrast to the United States where PCV13 is recommended among adults aged ≥ 65 years [23]. The JCVI decision acknowledges the extended benefit of the childhood PCV vaccination programme outside the targeted age groups as revealed by the serotype specific IPD surveillance and additional pneumococcal carriage and CAP studies.

Future research should focus on continued monitoring of the circulating vaccine-types. Should the projected declines in IPD and CAP incidence for the 6 additional serotypes in PCV13 are markedly different from the declines in disease due to the 7 serotypes in PCV7, revision of the above conclusions would be warranted.

## Supporting Information

S1 Appendix Comparison of the observed incidence of Community Acquired Pneumonia by Rodrigo et al. with the observed incidence in the CAPiTA trial and the observed incidence of J18 in the hospital. (DOCX)

S2 Appendix Comparison of the incidence rate ratio for invasive pneumococcal disease and pneumonia. (DOCX)

S3 Appendix Adjusting the population incidence for the non-risk population. (DOCX)
