# Supplementary material for: Cost-Effectiveness of Vaccinating Immunocompetent ≥65 Year Olds with the 13-Valent Pneumococcal Conjugate Vaccine in England
Source: PLoS One. 2016 Feb 25;11(2):e0149540. doi: 10.1371/journal.pone.0149540 (PMC4767406; doi:10.1371/journal.pone.0149540)
Supplement: S1 Appendix — (DOCX) [file pone.0149540.s001.docx]

**S1 Appendix Comparison of the observed incidence of Community Acquired Pneumonia by Rodrigo et al. with the observed incidence in the CAPiTA trial and the incidence of J18 in the hospital.**

In the analysis the incidence for CAP was based on the study by Rodrigo and colleagues [1]. To estimate the robustness of these a comparison was made between their findings and the observed incidence in the CAPiTA trial and in the Hospital Episode Statistics.

The applied case-definition to identify CAP and pneumococcal CAP was similar between Rodrigo et al. and the CAPiTA trial.

**Rodrigo et al.:** At least one of breathlessness, cough, sputum or fever, with new infiltrates on chest radiography consistent with pneumonia, and treated by the admitting clinical team for CAP. And for VT pneumococcal CAP: a positive vaccine-type–specific urinary antigen test or isolation of vaccine-type *S. pneumoniae* from blood or another sterile site.

**CAPiTA trial:** The presence of ≥2 prespecified clinical criteria and findings on chest radiography consistent with community-acquired pneumonia. And for VT pneumococcal CAP: a positive vaccine-type–specific urinary antigen test or isolation of vaccine-type *S. pneumoniae* from blood or another sterile site.

To compare the incidence in the CAPiTA trial with Rodrigo et al. the same age distribution as the CAPiTA trial was applied to re-weight the incidence observed by Rodrigo et al. We used the year 2008/2009 for comparison. Table 1 shows the incidence per 100,000 as observed in the trial and equivalent incidence observed by Rodrigo et al. Although Rodrigo et al. identified less CAP overall, the incidence of pneumococcal CAP and vaccine-type pneumococcal CAP was similar. Recently the incidence of inpatient CAP was also estimated for the Netherlands as part of a cost-effectiveness study [2], which estimated an incidence of 339 per 100.000 X-ray confirmed CAP when a similar age distribution was used as in the CAPiTA trial, which is somewhat in line with the observation of Rodrigo et al., in the paper is was subsequently assumed that 10% was caused by vaccine types, which will bring the VT-CAP incidence below both other estimates.

Table 1. Incidence per 100,000 person years, applying the age distribution of those at enrolment in the CAPiTA trial.

|  | CAPiTA | Rodrigo et al. year 08/09 |
| --- | --- | --- |
| Suspected CAP | 984 |  |
| Clinically confirmed CAP | 968 |  |
| CAP X-ray confirmed | 609 | 275 |
| Pneumococcal CAP (X-ray confirmed) | 110 | 100 |
| Vaccine type CAP (PCV13) | 67 | 67 |

To compare the incidence observed by Rodrigo at al. (2008/09) to the incidence of J18 (unspecified pneumonia) in the hospital, the incidence observed by Rodrigo et al for vaccine types was inflated to reflect all pneumococcal CAP (applying the distribution as observed in 2008/2009), and this was expressed as a percentage of the incidence of J18 observed by Trotter and colleagues [3]. See table 2. The percentage was around 20% which is in line with the literature. The percentage due to pneumococcus could however be up to around 40%.

Table 2. Comparison Rodrigo etal. with J18 in HES.

|  | Observed VT CAP Rodrigo 2008/09 | Percentage VT-types overall IPD 2008/09 | Projected pneumococcal CAP | Observed incidence J18 (Trotter et al) | Percentage pneumococcal CAP |
| --- | --- | --- | --- | --- | --- |
| 65-74 | 49.9 | 56% | 89.1 | 355 | 25% |
| 75-84 | 83.8 | 58% | 144.5 | 877 | 16% |
| 85+ | 281.3 | 54% | 520.9 | 2218 | 23% |

**Conclusion**, the incidence for VT-CAP reported by Rodrigo et al. is reasonable. There are some shortcomings in the presented comparisons, but the incidence seems in the same ballpark. Therefore a scenario was included that investigated a doubling of the CAP incidence, rather than tripling this incidence.

References:

[1] Rodrigo C, Bewick T, Sheppard C, et al. Impact of infant 13-valent pneumococcal conjugate vaccine on serotypes in adult pneumonia. Eur Respir J 2015;1–10.

[2] Mangen M-JJ, Rozenbaum MH, Huijts SM, et al. Cost-effectiveness of adult pneumococcal conjugate vaccination in the Netherlands. Eur Respir J 2015:ERJ – 00325–2015.

[3] Trotter CL, Stuart JM, George R, et al. Increasing hospital admissions for pneumonia, England. Emerg. Infect. Dis. 2008;14:727–33.
