# Supplementary material for: Cost-Effectiveness of Vaccinating Immunocompetent ≥65 Year Olds with the 13-Valent Pneumococcal Conjugate Vaccine in England
Source: PLoS One. 2016 Feb 25;11(2):e0149540. doi: 10.1371/journal.pone.0149540 (PMC4767406; doi:10.1371/journal.pone.0149540)
Supplement: S2 Appendix — (DOCX) [file pone.0149540.s002.docx]

**S2 Appendix Comparison Incidence rate ratio for invasive pneumococcal disease and pneumonia**

To compare the incidence risk ratios for invasive disease and pneumonia the IPD incidence as observed by PHE in its surveillance data set for England and Wales was used with 2008/09 as base year, as well as the incidence for CAP as observed by Rodrigo et al. with the same base year. For both datasets the information was split between the serotypes covered by PCV7 and PCV13 minus PCV7 types. The decline in vaccine type IPD and CAP is similar.

Figure A1 The Incidence risk ratio for IPD and CAP as observed by PHE and Rodrigo et al. The black lines represent IPD, and the grey CAP. Panel A shows the PCV7 types, panel B the PCV13 minus PCV7 types

A)

**
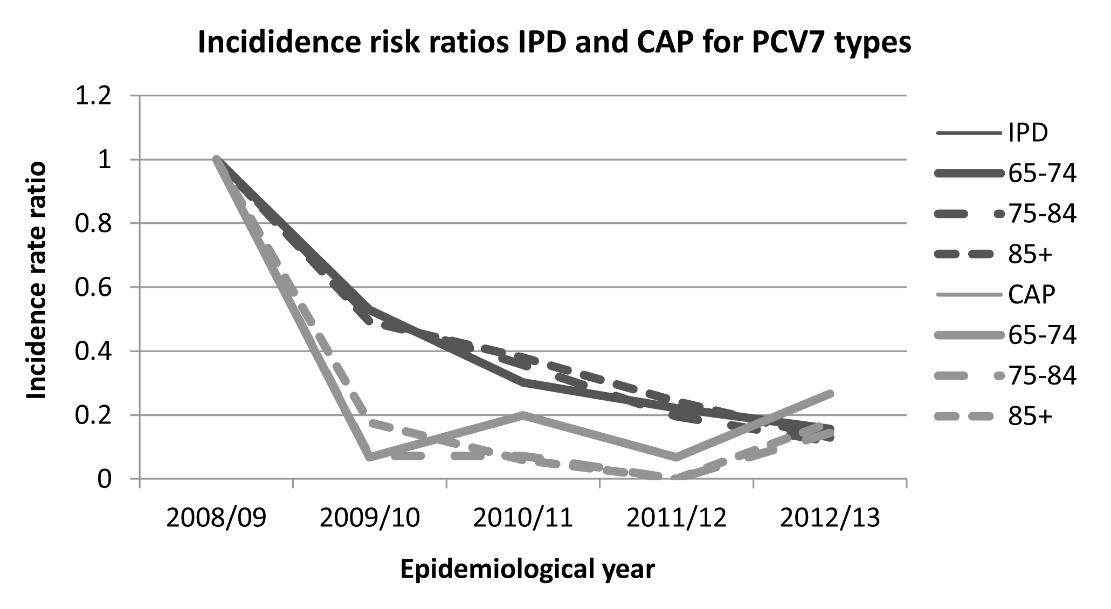
**

B)

**

**
