# Supplementary material for: Cost-Effectiveness of Vaccinating Immunocompetent ≥65 Year Olds with the 13-Valent Pneumococcal Conjugate Vaccine in England
Source: PLoS One. 2016 Feb 25;11(2):e0149540. doi: 10.1371/journal.pone.0149540 (PMC4767406; doi:10.1371/journal.pone.0149540)
Supplement: S3 Appendix — (DOCX) [file pone.0149540.s003.docx]

**S3 Appendix Adjusting the population incidence for the non-risk population**

The incidence in the non-risk population expressed as a percentage of the overall incidence (55%) was obtained as follows. Assuming that 45% of those aged 65 and older were not in a clinical risk group (non-risk) [4] and that the odds ratio for those within a risk group was 2.8 [4] allowed for a redistribution of the IPD cases among risk groups and those in a clinical risk-groups such that none of the assumptions were violated. This resulted in an estimated 30.4% of IPD cases being among the non-risk group population or that the relative incidence among those not in a risk group should be 55% of the incidence observed in the overall population (non-risk + risk group). Below is a representation of the calculation for an example with a population incidence of 2.3 per 100,000.

Table 1. Redistribution of IPD cases among those not in a risk group and those who are, such that the odds ratio for those in a risk group was 2.8. This shows that the incidence among non-risk groups is 55% of the total population estimate.

|  | Non-risk | Clinical Risk | Total | Odds +Odds ratio |
| --- | --- | --- | --- | --- |
| Population # | 352,666 (55%) | 288,545 (45%) | 641,210 (100%) | 0.82 (CR/NR) |
| IPD # | 4.49  (30.4%) | 10.29  (69.6%) | 14.78  (100%) | 2.29 (CR/NR) |
| Incidence (per 100,000) | 1.27 | 3.57 | 2.3 | OR= 2.8 (IPD/Population) |
|  | 55% | 45% | 100% |  |
